# Medication adherence in pregnant women with human immunodeficiency virus receiving antiretroviral therapy in sub-Saharan Africa: a systematic review

**DOI:** 10.1186/s12889-018-5651-y

**Published:** 2018-06-27

**Authors:** Olumuyiwa Omonaiye, Snezana Kusljic, Pat Nicholson, Elizabeth Manias

**Affiliations:** 1 Centre for Quality and Patient Safety Research, School of Nursing and Midwifery, Faculty of Health, Deakin University, 221 Burwood Highway, Burwood Campus, Melbourne, VIC 3125 Australia; 2 Department of Nursing, School of Health Sciences, Faculty of Medicine, Dentistry and Health Sciences, The University of Melbourne, Melbourne, Australia

**Keywords:** HIV, Adherence, Prevention of mother-to-child transmission, Antiretroviral therapy, Pregnant women, Sub-Saharan Africa

## Abstract

**Background:**

The use of antiretroviral therapy (ART) is a core strategy proposed by the World Health Organization in preventing mother to child transmission (MTCT) of HIV. This systematic review aimed to examine the enablers and barriers of medication adherence among HIV positive pregnant women in sub-Saharan Africa.

**Methods:**

We used the following keywords: HIV AND (Pregnancy OR Pregnant*) AND (PMTCT OR “PMTCT Cascade” OR “Vertical Transmission” OR “Mother-to-Child”) AND (Prevent OR Prevention) AND (HAART OR “Antiretroviral Therapy” OR “Triple Therapy”) AND (Retention OR Concordance OR Adherence OR Compliance) to conduct electronic searches in the following databases: MEDLINE Complete (1916-Dec 2017), Embase (1947-Dec 2017), Global Health (1910-Dec 2017) and CINAHL Complete (1937-Dec 2017). Of the four databases searched, 401 studies were identified with 44 meeting the inclusion criteria. Seven studies were added after searching reference lists of included articles, resulting in 51 articles in total.

**Results:**

The review demonstrated that stigma, cost of transportation, food deprivation and a woman’s disclosure or non-disclosure of her HIV status to a partner, family and the community, could limit or define the extent of her adherence to prescribed antiretroviral drugs during pregnancy. Furthermore, the review indicated that knowledge of HIV status, either before or during pregnancy, was significantly associated with medication adherence. Women who knew their HIV status before pregnancy demonstrated good adherence while women who found out their HIV infection status during pregnancy were linked with non-adherence to ART.

**Conclusion:**

This review revealed several barriers and enablers of adherence among pregnant women taking ART in sub-Saharan Africa. Major barriers included the fear of HIV infection status disclosure to partners and family members, stigma and discrimination. A major enabler of adherence in women taking ART was women’s knowledge of their HIV status prior to becoming pregnant. Enhanced effort is needed to facilitate women’s knowledge of their HIV status before pregnancy to enable disease acceptance and management, and to support pregnant women and her partner and family in dealing with fear, stigma and discrimination about HIV.

## Background

Living with human immunodeficiency virus (HIV) is a major health concern in sub-Saharan Africa. In 2015, more than 90% of the world’s 1.4 million pregnant women with HIV lived in sub-Saharan Africa, with 160,000 new HIV infections among children aged 0–14 years reported in 2016 [1, 2]. Over 90% of cases involving HIV infection in children are linked to mother-to-child transmission (MTCT) [3] and without intervention, the risk of MTCT ranges between 25 and 48% [4, 5]. However, with specific interventions, particularly with the use of antiretroviral therapy (ART), transmission rates can be reduced to less than 2 and 5% in non-breastfeeding and breastfeeding populations respectively [3]. Mother-to-child vertical transmission of HIV can occur during three major periods; antenatally, around the time of delivery or birth, called intrapartum, and postpartum, which is attributable to breastfeeding [6]. About 80% of MTCT is believed to take place during the intrapartum period.

The “90–90-90” United Nations (UNAIDS) agenda, launched in 2014, proposes that 90% of all people living with HIV will know their HIV status, 90% of all people with diagnosed HIV infection will receive sustained antiretroviral therapy and 90% of all people receiving antiretroviral therapy will have viral suppression by 2020 [7].The 2017 progress report by UNAIDS showed eastern and southern Africa has made huge strides towards meeting the 90–90–90 targets [8]. Three out of four people living with HIV in these regions are aware of their HIV status, nearly four in five who know their HIV status are on treatment, and more than four in five who are on treatment have suppressed viral loads [8]. On the other hand, in western and central Africa, HIV testing and treatment coverage are far below the global average [8]. Therefore, to reach the targets in the context of MTCT of HIV and paediatric care of HIV, it is critical to scale up HIV testing and provision of ART for pregnant women and children living with HIV in sub-Saharan Africa [9].

Given that sub-optimal adherence has been reported with a short course of ART prophylaxis in most countries in sub-Saharan Africa [8–10], there is a concern that adherence levels may further plummet in view of the current WHO guidelines (option B+). These guidelines recommend that individuals should take triple-drug ART for life [10]. Nevertheless, it is crucial that medication adherence is optimal during pregnancy when peak periods of HIV transmission are high in order to achieve maximum viral load suppression and prevent MTCT [10]. Preventing MTCT of HIV is likely to be a challenge, especially in resource-poor settings where women have limited access to an elective caesarean section and viral load monitoring [11–13]. The aim of this review is to examine barriers and enablers of medication adherence to ART among HIV positive pregnant women in sub-Saharan Africa.

## Methods

### Study eligibility

#### Inclusion criteria

The preferred reporting items for systematic review and meta-analysis (PRISMA) guideline was used to facilitate the systematic review process [14]. Quantitative and qualitative studies conducted in sub-Saharan Africa, involving HIV positive pregnant women and focusing on medication adherence to ART, were included in the review. No restrictions were placed on language and publication date.

#### Exclusion criteria

**Fig. 1:**
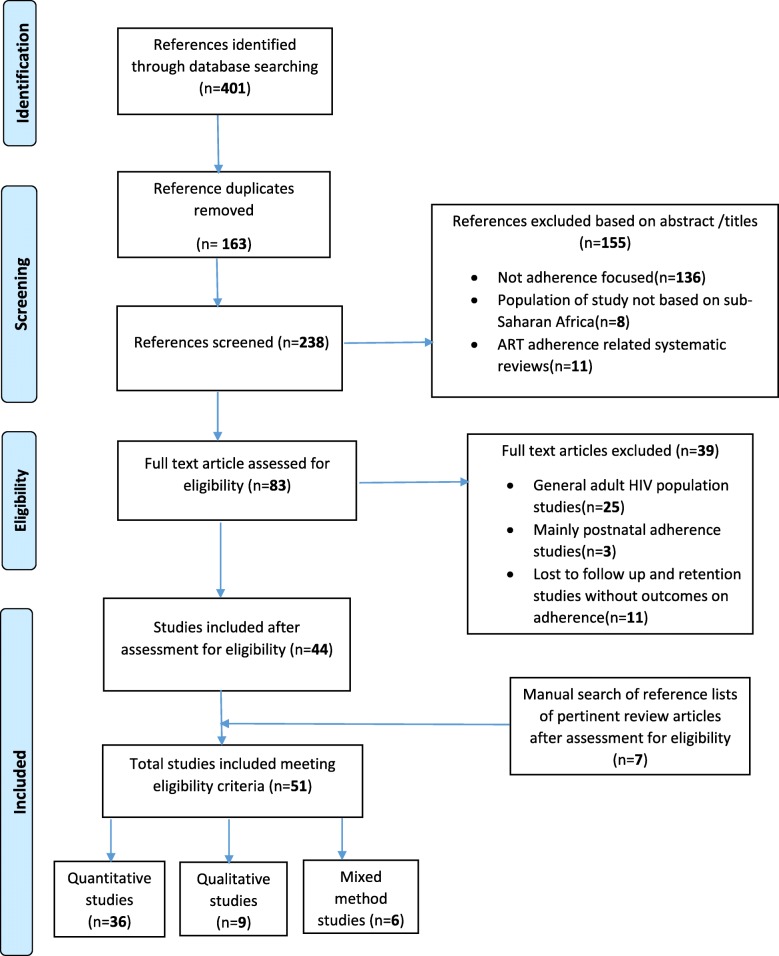
PRISMA flow diagram of systematic search results and study selection

Studies were excluded if they involved medication adherence in HIV positive postpartum mothers. Commentaries, editorials, reviews and letters were also excluded. Figure 1 shows the PRISMA diagram depicting the search process, screening, eligibility, and inclusion and exclusion procedures.

### Information sources and search strategy

We used the following keywords to search the databases: HIV AND (Pregnancy OR Pregnant*) AND (PMTCT OR “MTCT” OR “Vertical Transmission” OR “Mother-to-Child”) AND (Prevent OR Prevention) AND (HAART OR “Antiretroviral Therapy” OR “Triple Therapy”) AND (Retention OR Concordance OR Adherence OR Compliance). The following electronic databases were used: MEDLINE Complete (1916-December 2017), Embase (1947-December 2017), Global Health (1910-December 2017) and CINAHL Complete (1937-December 2017). Articles obtained from these searches were imported into EndNote (X7 version) and duplicates were removed.

### Study selection

One researcher (O.O.) initiated and screened all titles and abstracts to identify potentially relevant studies. A second researcher independently screened all titles and abstracts to identify potentially relevant studies (E.M.). The full texts of potentially relevant studies were independently examined by three researchers (O.O., E.M. and S.K.) to determine whether they met the inclusion criteria. In addition, O.O., E.M and S.K. independently manually searched included papers to seek out additional articles for inclusion. If there was uncertainty about whether certain studies met the inclusion criteria, negotiated agreement through consensus was reached by the researchers.

### Data extraction

Once the study selection process was completed, one of the authors (O.O) used a standard template [15] to document information from the selected studies. This information included the population studied, setting, research design, participants, sample size, and data collection methods used to assess adherence. Information relating to barriers and enablers of medication adherence was also documented from studies. Independent review of data abstraction process was conducted by E.M., S.K. and P.N. and discrepancies resolved by discussion.

### Risk of bias and quality assessment of studies

Two researchers independently assessed the risk of bias and quality of included studies using the Mixed Method Appraisal Tool (MMAT) version 2011, which provides criteria to evaluate the methodological quality of studies [16]. The MMAT tool was chosen because it has been validated for use in studies involving diverse research designs [17, 18]. The MMAT comprises 19 items assessing the quality and possible risk of bias of five different types of studies (qualitative research, randomized controlled trials (RCTs), non-randomized studies, quantitative descriptive studies, and mixed methods studies). Each item is rated as “yes,” “no,” or “cannot tell” for each applicable item.

### Categorization of enablers and barriers of adherence to ART

According to the WHO, adherence to long term therapies is a complex phenomenon that is influenced by the interaction of five broad dimensions [19], which comprises the WHO multidimensional adherence model [19]. Categorization of enablers and barriers of adherence in this review is based on this model. The five dimensions are: patient related factors, condition (factors related to patient symptoms, level of disability, severity of disease, rate of progress of the disease, presence of co-morbidities), therapy related factors (the complexity of the therapy, the immediacy of beneficial effects, and side-effects), social and economic related factors, and health care team and health system related factors. Tables 1 and 2 provide detailed information on these five dimensions.

**Table 1:** Themes of adherence to medication among HIV positive pregnant women included in qualitative studies (*N* = 15)

Domain	Themes	References (study numbers)
Patient	Psychological	
	Shock/denial following a positive HIV result	[1, 6, 9, 14]
	Motivation to protect infant/self/family	[6, 9, 10]
	Patient knowledge on ART/PMTCT	
	Poor knowledge of ART, MTCT	[4]
	Good knowledge of ART	[3]
	Self-efficacy	
	Self-reported ability to adhere to ART	[6, 8]
	Patient attitude and personal management	
	Late ANC attendance an obstacle to early AZT prophylaxis and initiation on HAART	[2]
	Missing clinical appointments	[1]
	Interrupted personal routine	[4]
	Patient belief system	
	Religious belief	[13]
	Use of traditional medicines	[5]
Patient condition	Disease progression	[13]
	Obstetric/ pregnancy	
	Uncertainty about onset of labour in order to swallow NVP	[1]
	Previous experience with PMTCT	[9]
Therapy	Side effects of ART	[6, 13]
	Perceived effectiveness of therapy	[6, 9]
Social and economic factors	Financial difficulty	
	Lack of transport fee to go to health facility for pick-up of ARVs	[1, 2, 3, 13]
	Lack of money to buy food to eat	[3, 4, 13]
	Women empowerment	
	Economically dependent on husbands	[3]
	Domestic violence either actual or threatened	[4]
	Lack of male involvement	[2]
	Cultural conditions and beliefs	
	Traditional medicines/healers	[4, 5]
	Religious beliefs	[13]
	Partner and community – the challenges of disclosure and non-disclosure	
	Fear of disclosure to partner/family members	[3, 4, 5, 8, 9, 10, 13, 14]
	Disclosure to partner/family(facilitating)	[8, 13]
	HIV infected relatives stealing tablets	[4]
	Hiding ARVS within the house/taking ARVs in hiding in the house	[4, 5]
	Pattern of misinformation on ART in the community	[14]
	HIV related stigma	[2, 5, 9, 10, 13, 15]
	Community view of HIV- infected persons had no bearing on their decision to begin or continue ART	[3]
	Sharing medication with others	
	Sharing ARVs with partner/friend	[1, 4]
	Positive outlook of known patients living with HIV in the community	
	Seeing positive results in the community of women taking ART and looking healthy	[9, 10, 12]
Health care team/health system	Staff related	
	Fear of mistreatment by HCWs	[1, 3, 7, 10]
	HCWs providing good counselling on ART	[6, 7, 8]
	Supply chain management system	
	Delayed supply of ARVs	[2, 6, 7]
	Resource/Infrastructure and service related	
	Prolonged counselling to initiate prophylaxis or ART	[2, 3]
	Lack of privacy and confidentiality	[2, 7]
	Inadequate counselling and short contact time with patient	[2]
	Long waiting time in the health facilities	[3, 11]

*ANC* Antenatal clinic, *HCWs* Health care workers, *ARV* Antiretroviral, *ART* Antiretroviral therapy, *MTCT* Mother to child transmission of HIV, *NVP* Nevirapine, *AZT* Zidovudine. Study numbers = the order in which the references appear on Table 4

**Table 2:** Predictors of adherence to medication among HIV positive pregnant women included in the quantitative studies

Domain	Predictors	Total number with statistical evidence for association	*List of citations with statistical evidence for association	Total number without statistical evidence for association	List of citations with no statistical evidence for association
Patient	Socio-demographic				
	Age of mother	3	Igwegbe, et al. 2010 [34], Parisotto, et al. 2011 [33], Haas, et al. 2016 [32].	13	Kiarie, et al. 2003 [30], Delvaux, et al. 2009 [29], Bancheno, et al. 2010 [28], Peltzer, et al. 2010 [27], Kinuthia, et al. 2011 [26], Kirsten, et al. 2011 [25], Mirkuzie, et al. 2011 [24], Ekama, et al. 2012 [31], Buseri, et al. 2014 [23], Hampanda 2016 [21], Okawa, et al. 2015 [22], Schnack, et al. 2016 [10], Yotebieng, et al. 2016 [20].
	Married/living with partner	1	Delvaux, et al. 2009 [29].	9	Kiarie, et al. 2003 [30], Igwegbe, et al. 2010 [34], Peltzer, et al. 2010 [27], Shapiro, et al. 2010 [66], Kinuthia, et al. 2011 [26], Ekama, et al. 2012 [31], Okawa, et al. 2015 [22], Schnack, et al. 2016 [10], Yotebieng, et al. 2016 [20].
	Education	5	Delvaux, et al. 2009 [29], Igwegbe, et al. 2010 [34], Kuonza*,* et al. 2010 [36], El-Khatib, et al. 2011 [35], Schnack, et al. 2016 [10].	8	Kiarie, et al. 2003 [30], Bancheno, et al. 2010 [28], Peltzer, et al. 2010 [27], Kinuthia, et al. 2011 [26], Kirsten, et al. 2011 [25], Mirkuzie, et al. 2011 [24], Hampanda 2016 [21], Okawa, et al. 2015 [22]
	Illiteracy in primary language	1	Stringer, et al. 2003 [37].	0	
	Patient belief system				
	Religion	0		1	Delvaux, et al. 2009 [29].
	Cultural beliefs: Use of traditional medicine/visiting traditional healer/birth attendant	2	Banda, et al. 2007 [47], Itoua, et al. 2015 [46].	2	Kuonza*,* et al. 2010 [36], Peltzer, et al. 2011 [50].
	Patient knowledge about HIV/MTCT	2	Peltzer, et al. 2010 [27], Ekama, et al. 2012 [31].	3	Peltzer, et al. 2008 [52], Peltzer, et al. 2011 [50], Ebuy, et al. 2015 [48].
	Patient knowledge of HIV diagnosis before pregnancy	1	Parisotto, et al. 2011 [33].	1	Igwegbe, et al. 2010 [34].
	Patient knowledge of HIV diagnosis during pregnancy	2	Igwegbe, et al. 2010 [34], Okawa, et al. 2015 [22].	0	
	Patient attitude to MTCT	0		2	Ebuy, et al. 2015 [48], Hampanda 2016 [21].
	Psychological				
	Forgetting to take ARV	1	Itoua, et al. 2015 [46].	0	
Patient condition	Obstetric/ pregnancy history				
	Parity	2	Igwegbe, et al. 2010 [34], Kuonza*,* et al. 2010 [36].	5	Kiarie, et al. 2003 [30], Kirsten, et al. 2011 [25], Mirkuzie, et al. 2011 [24], Ekama, et al. 2012 [31], Hampanda 2016 [21].
	Cervical dilation	1	Megazzini, et al. 2009 [58]	0	
	Stage of pregnancy/gestational age	2	Barigye, et al. 2010 [57], Parisotto, et al. 2011 [33].	4	Mirkuzie, et al. 2011 [24], Ebuy, et al. 2015 [48], Schnack, et al. 2016 [10], Yotebieng, et al. 2016 [20].
	Exposed to PMTCT in previous pregnancy	1	Kuonza*,* et al. 2010 [36].	2	El-Khatib, et al.2011 [35], Ekama, et al. 2012 [31]
	Premature delivery	1	Peltzer, et al. 2010 [27].	0	
	WHO Clinical staging	2	Ebuy, et al. 2015 [48], Haas, et al. 2016 [32].	0	
	CD4 count at admission	1	Ebuy, et al. 2015 [48],	0	
Therapy	Duration on therapy	2	Barigye, et al. 2010 [57], Igwegbe, et al. 2010 [34].	1	Mirkuzie, et al. 2011 [24].
	Type of PMTCT drug regimen	1	Ebuy, et al. 2015 [48].	1	Mepham, et al. 2011 [38]
	ARV side effects	1	Kamuyango, et al. 2014 [77]	1	Itoua, et al. 2015 [46].
Social and economic	Women empowerment				
	Not receiving financial support from a partner or husband	1	El-Khatib, et al. 2011 [35].	0	
	Income generating activities /occupation	2	Igwegbe, et al. 2010 [34], Bisio, et al.2013 [75].	7	Kiarie, et al. 2003 [30], Kuonza*,* et al. 2010 [36], El-Khatib, et al. 2011 [35], Kinuthia, et al. 2011 [26], Kirsten, et al. 2011 [25], Ekama, et al. 2012 [31], Hampanda 2016 [21], Schnack, et al. 2016 [10].
	Housing(monthly rent)	1	Igwegbe, et al. 2010 [34].	0	
	Financial difficulty				
	Distance to health facility	0		2	Itoua, et al. 2015 [46], Schnack, et al. 2016 [10].
	Partner and community				
	Partner aware/informed of HIV test results/willing to have HIV test/partner came for HIV test/partner support use of ARV	10	Kiarie, et al. 2003 [30], Farquhar, et al. 2004 [54], Msuya, et al. 2008 [53], Peltzer, et al. 2008 [52], Delvaux, et al. 2009 [29], Igwegbe, et al. 2010 [34], Peltzer, et al. 2010 [27], Kirsten, et al. 2011 [25], Ebuy, et al. 2015 [48], Hampanda 2016 [21].	4	Barigye, et al. 2010 [57], Kinuthia, et al. 2011 [26], Mepham, et al. 2011 [38], Mirkuzie, et al. 2011 [24].
	Partner uninformed about ARV	1	Itoua, et al. 2015 [46].	1	Matthews, et al. 2016. [76]
	Disclosure to someone(not partner)/discussion of PMTCT prophylaxis with family/friends/others	6	Peltzer, et al. 2008 [52], Delvaux, et al. 2009 [29], Peltzer, et al. 2010 [27], Peltzer, et al. 2011 [50], Ekama, et al. 2012 [31], Ebuy, et al. 2015 [48].	3	Barigye, et al. 2010 [57], Mepham, et al. 2011 [38], Matthews, et al. 2016 [76].
	male partner involvement in PMTCT/ANC	2	Peltzer, et al. 2011 [50], Ebuy, et al. 2015 [48].	1	Schnack, et al. 2016 [10].
	Partner violence				
	Sexual violence	1	Hampanda 2016 [21].	0	
	Emotional violence	0		1	Hampanda 2016 [21].
	Physical violence	0		1	Hampanda 2016 [21].
	Partners’ HIV status				
	Positive	1	Igwegbe, et al. 2010 [34].	0	
	Negative	0		1	Igwegbe, et al. 2010 [34].
	Unknown	0		1	Igwegbe, et al. 2010 [34].
	Attend support group/treatment supporter/partner support	4	Peltzer, et al. 2010 [27], Peltzer, et al. 2011 [50], Ekama, et al. 2012 [31], Ebuy, et al. 2015 [48].	0	
	Place of residence				
	Living in a village where HIV research is being conducted	1	Barigye, et al. 2010 [57].	0	
	Living in rural or urban.	0		1	Ebuy, et al. 2015 [48]
	HIV related stigma/experienced discrimination	3	Peltzer, et al. 2010 [27], Kinuthia, et al. 2011 [26], Peltzer, et al. 2011 [50].	0	
	Feeling embarrassed	1	Itoua, et al. 2015 [46].	0	
Health care team/Health system	Number of ANC visits/clinic attendance	3	Peltzer, et al. 2010 [27], Megazzini, 2009 [58], Ebuy, et al. 2015 [48]	1	Mepham, et al. 2011 [38].
	Quality of post-test counseling/counseling on ARV side effects	2	Peltzer, et al. 2010 [27], Ebuy, et al. 2015 [48].	0	
	Confidentiality at health facility	1	Peltzer, et al. 2010 [27]	0	

*ANC* Antenatal clinic, *ARV* Antiretroviral, *ART* Antiretroviral therapy, *MTCT* Mother to child transmission of HIV, *WHO* World Health Organization, *PMTCT* Prevention of mother to child transmission of HIV:*Statistical evidence for an association (*p* < 0.05 or90%CI excludes the null value of one) was reported with at least one relevant/related outcome such as adherence to medication/receipt or ingestion PMTCT ARVs for prophylaxis/combination ART/attendance at ART/PMTCT clinic

## Results

### Characteristics of included studies

The systematic search yielded 401 articles, of which 44 met the inclusion criteria. An additional 7 were added after screening references, resulting in 51 included articles (Fig. 1). Of the 51 articles, 36 were quantitative, 9 were qualitative and 6 were mixed methods studies. Tables 3 and 4 provide detailed information on characteristics of included studies.

**Table 3:** Characteristics of quantitative studies included (*N* = 42)

S/N	Author: Year	Country: Setting	Study Design	Participants/overall sample size	Type of PMTCT intervention implemented	QAR of studies
1	Meda, et al. 2002 [64]	Cote’d Ivoire and Burkina Faso Urban in both countries.	Randomised clinical trial	HIV+ pregnant women enrolled in an open label cohort at 36–38 weeks gestation to receiving an oral course of zidovudine. (404)	ZDV	**
2	*Kiarie, et al. 2003 [30]	Kenya: Urban	Randomised clinical trial	HIV+ pregnant women enrolled in a clinical trial in a tertiary HF. (124)	ZDV,SdNVP	****
3	Stringer, et al. 2003 [37]	Zambia: Urban	Clustered-randomised clinical trial	HIV+ pregnant women attending two HF in the trial. (201)	SdNVP	**
4	Farquhar, et al. 2004 [54]	Kenya: Urban	Prospective cohort study	Pregnant women attending one clinic; male partners.(1991)	SdNVP	****
5	Banda, et al. 2007 [47]	Zambia: Urban	Clinical trial: sub-analysis	HIV + pregnant women enrolled in a clinical trial of perinatal HIV prevention strategies at two district delivery centres.(78)	SdNVP	*
6	Coffie,et al. 2008 [69]	Ivory Cost: Urban	Prospective cohort study	HIV + women pregnant registered in a PMTCT plus programme. (247)	cART (ZDV + 3TC + NVP) (94.7%): (D4T + 3TC + NVP) (2.8%): (ZDV + 3TC + EFV (2.1%): (D4T + 3TC + EFV) (0.4%).	***
7	Msuya, et al. 2008 [53]	Tanzania: Urban	Prospective cohort study	HIV+ pregnant women attending ANC at two public clinics for PMTCT services; with/without male partner support. (184)	SdNVP	****
8	Peltzer, et al. 2008 [52]	South Africa: Unclear	Cross sectional study	HIV+ pregnant women in a PMTCT cohort from five clinics (66).	SdNVP	****
9	Delvaux, et al. 2009 [29]	Rwanda: Urban and rural	Case-control study	HIV + pregnant women who did not adhere (cases) and adhered (control) to PMTCT prophylaxis at 12 PMTCT sites. (236)	SdNVP	****
10	Megazzini, et al. 2009 [58]	Zambia: Urban	Clustered randomised clinical trial: sub-analysis	Pregnant women in the trial intervention arm who had HCT in the labour ward.(23)	SdNVP	*
11	*Bancheno, et al. 2010 [28]	Swaziland: Rural	Retrospective cohort study	HIV + pregnant women registered for PMTCT services in a rural HF. (99)	SdNVP	*
12	Barigye, et al. 2010 [57]	Uganda: Rural	Prospective cohort study	HIV + pregnant women registered for PMTCT services at four clinics located in study and non-study villages. (83)	SdNVP	****
13	Igwegbe, et al. 2010 [34]	Nigeria: Urban	Cross sectional study	HIV+ pregnant women attending PMTCT clinic in a tertiary HF.(368)	cART (specific ARV drugs combination NR).	***
14	Kuonza*,* et al. 2010 [36]	Zimbabwe: Urban	Cross sectional study	HIV + pregnant women and their infants registered in PMTCT programme in four HF. (212)	SdNVP	***
15	Megazzini, et al. 2010 [65]	Zambia: Urban	Clustered randomised clinical trial:	HIV + pregnant women receiving PMTCT services in 12 public delivery centres. (498)	SdNVP	*
16	Peltzer, et al. 2010 [27]	South Africa: Rural	Cross sectional study	HIV + pregnant women and their infants at 47 HFs. (815)	SdNVP	***
17	Shapiro, et al. 2010 [66]	Botswana: Urban and rural	Randomised control trial	HIV + pregnant women enrolled into the trial at four HFs.(730)	cART (ABC + AZT + 3TC): Lopinarvir-ritonavir+AZT + 3TC): (NVP + AZT + 3TC).	**
18	El-Khatib,et al. 2011 [35]	South Africa: Urban	Prospective cohort study	HIV + women pregnant registered in an ART programme in a single HF. (147)	cART (D4T + 3TC + NVor EFV).	***
19	Kinuthia, et al. 2011 [26]	Kenya: Urban	Cross sectional study	Sub set of HIV+ women and their infants attending six MCH clinics.(318)	Short course AZT regimen, sdNVP.	****
20	Kirsten, et al. 2011 [25]	Tanzania: Rural	Prospective cohort study	HIV + women pregnant registered in a single HF offering PMTCT services. (150)	AZT and cART (AZT + 3TC).	****
21	*Mepham, et al. 2011 [38].	South Africa: Rural	Randomised control trial	HIV + pregnant women receiving PMTCT services in a HF. (94)	cART(specific ARV drugs combination NR).	*
22	Mirkuzie, et al. 2011 [24]	Ethiopia: Urban	Prospective cohort study	HIV + pregnant women attending 15 HF and their infants.(282)	cART and ZDV prophylaxis.	***
23	Parisotto, et al. 2011 [33]	Burkina Faso: Urban	Retrospective cohort study	HIV + pregnant women and their children at one HF.(229)	Short course ARV prophylaxis: Actual ARV drug(s) NR.	****
24	Peltzer, et al. 2011 [50]	South Africa: Rural	Cross sectional study	HIV + pregnant and postnatal women and their infants recruited from 48 HFs.(746)	AZT	****
25	Ekama, et al. 2012 [31]	Nigeria: Urban	Cross sectional study	HIV+ pregnant women registered for PMTCT services at ART treatment centre.(170)	cART (specific ARV drugs combination NR).	**
26	Okonji, et al.2012 [67]	Kenya: Urban	Clinical trial: sub-analysis	HIV + women pregnant enrolled into the trial at a single HF rendering combination ART for PMTCT.(434)	cART (NVP + 3TC + ZDV, NFV + 3TC + ZDV).	**
27	Bisio, et al. 2013 [75]	Republic of Congo: Urban and rural.	Prospective uncontrolled interventional study	HIV + women pregnant attending four Antenatal clinics.(415)	cART(AZT + 3TC + NVP, D4T + 3TC + NVP)	***
28	Denoeud-Ndam*,* et al. 2013 [70]	Benin: Urban	Prospective cohort study	HIV + women pregnant at 5 hospitals attending ANC. (217)	cART (EFV/NVP/PI).	***
29	Buseri, et al. 2014 [23]	Nigeria: Urban and rural.	Cross sectional study	HIV + pregnant women receiving PMTCT services at ANC in four hospitals. (312)	cART (AZT or D4T + 3TC + NVPor EFV).	**
30	Kamuyango et al. 2014 [77]	Malawi: Urban and rural	Retrospective cohort study	HIV + women in ART sites who received ARV intervention for PMTCT.(292)	cART (D4T + 3TC + NVP: TDF + 3TC + EFV).	****
31	*Murithi, et al. 2015 [41]	Kenya: Urban	Cross sectional study	HIV + women pregnant receiving care in a PMTCT clinic.(55)	cART(specific ARV drugs combination NR).	**
32	Ebuy, et al. 2015 [48]	Ethiopia: Urban and rural	Cross sectional study	HIV + women pregnant registered for PMTCT in six hospitals. (263)	cART (EFV + 3TC + TDF).	****
33	Itoua,et al. 2015 [46]	Republic of Congo: Urban	Cross analytical and transversal study	HIV + women pregnant and lactating enrolled in three treatment centres. (80)	cART (specific ARV drugs combination NR).	**
34	Ngoma, et al. 2015 [71]	Zambia: Urban	Prospective cohort study	HIV + pregnant women with the ability to initiate combination ART AT 14–30 weeks at a public HF. (226)	cART (AZT + 3TC + Lopinavir/ ritonavir).	***
35	Okawa, et al.2015 [22]	Zambia: Rural	Prospective cohort study	HIV + pregnant women registered in PMTCT service at eleven HFs. (481)	AZT, sdNVP, and cART (specific ARV drugs NR).	***
36	*Granato, et al. 2016 [78]	Cote d’ Ivoire: Urban and Rural.	Cross sectional study	HIV positive pregnant women enrolled in PMTCT services attending 29 HFs. (219)	cART (specific ARV drugs combination NR).	****
37	Haas, et al. 2016 [32]	Malawi: Urban and rural.	Retrospective cohort study	HIV + women pregnant and postpartum women who commenced ART regimen at 13 HFs. (4248)	cART(EFV + 3TC + TDF	****
38	Hampanda 2016 [21]	Zambia: Urban	Cross sectional study	HIV + postpartum women and their infants at a large HF attending child immunization.(402)	SdNVP and cART (specific ARV drugs combination NR).	****
39	Matthews, et al. 2016 [76]	Uganda: Rural	Prospective cohort study	HIV + pregnant women; a subset of more than 7000 HIV infected adults initiating their ART in a tertiary HF.(396)	cART (specific ARV drugs combination NR).	****
40	*Napua, et al. 2016 [40]	Mozambique: Urban	Clustered(facility level) randomised controlled trial	HIV + women pregnant registered in 6 high volume HFs providing PMTCT and ART services. (141)	cART (specific ARV drugs combination NR).	*
41	Schnack, et al. 2016 [10]	Uganda: Urban.	Observational longitudinal study.	HIV + pregnant women attending ANC in two hospitals with PMTCT programme. (76)	cART (specific ARV drugs combination NR).	**
42	Yotebieng, et al. 2016 [20]	DRC Congo: Urban.	Randomised clinical trial	Newly diagnosed HIV + pregnant women registering for ANC at 89 HFs. (300)	AZT, and cART (specific ARV drugs combination NR).	*

*Quantitative aspect of a mixed method study: S/N also indicate study number, the sequential order of articles; differs from the bibliographic reference number: *QRA* Quality rating of studies, *cART* Combination antiretroviral therapy(triple drug combination), *NR* Not reported, *HF* Health facility, *sdNVP* Single dose nevirapine, *PMTCT* Prevention of mother to child transmission of HIV, *MCH* Maternal and child health, *PLA* Participatory learning and action, *HCWs* Health care workers, *DRC* Democratic republic of Congo, *ANC* Antenatal clinic: AZTor *ZDV* Zidovudine, *TDF* Tenofovir, *NFV* Nelfinavir, *3TC* Lamivudine, *D4T* Stavudine, *EFV* Efavirenz, *ABC* Abacavir, *ART* Antiretroviral therapy: Quality assessment rating of studies: using the mixed methods appraisal tool an overall quality score of (*) in one of the four methodological assessment area represent 25%; (****) = all four criteria met

**Table 4:** Characteristics of qualitative studies included (*N* = 15)

S/N	Author: Year	Country: Setting	Study design	Participants and type of PMTCT intervention implemented	Sample size	QAR of studies
1	*Kiarie, et al. 2003 [30]	Kenya: Urban	FGDs	HIV+ pregnant and postpartum women enrolled in a clinical trial in a tertiary HF. (ZDV,SdNVP)	7 FGDs (4 sessions with pregnant women and 3 sessions with postpartum women).	****
2	*Bancheno, et al. 2010 [28]	Swaziland: Rural	IDIs	HIV + pregnant women registered for PMTCT services in a rural HF: Nurses, nurse assistants. (SdNVP)	64 Interviews (nurses and nurse assistants)	***
3	Duff, et al.2010 [39]	Uganda: Urban and rural	IDIs and FGDs	HIV + mothers registered in PMTCT programme. (cART)	45 IDIs; 1 FGD (8 women).	****
4	*Mepham, et al. 2011 [38].	South Africa: Rural	IDIs	HIV + pregnant women receiving PMTCT services in a HF. (cART)	20 interviews	****
5	Ujiji, et al. 2011 [45]	Kenya: Rural	Narratives	HIV + pregnant women already receiving ART in two HFs. (cART)	28; 12 urban and 16 rural.	***
6	Buesseler, et al. 2014 [42]	Cote d’Ivoire:Rural	IDIs	HIV + mothers registered in PMTCT integration programme at four HFs: HCWs. (cART)	IDIs:24 women,5 HCWs.	***
7	Gourlay, et al. 2014 [59]	Tanzania: Rural	PLA and IDIs	HIV + women attending four HF for PMTCT services: HIV- women: HCWs: Health officials. (specific short course antiretroviral regimen not mentioned)	61 PLA (3male, 3 female groups with 8–12 participants): 30 IDIs (16 HIV + &5 HIV – women, 6 HCWs, 3 health officials.	****
8	Kastner, et al. 2014 [51]	Uganda: Urban	IDIs	Women attending HIV clinic for follow up (HIV + pregnant women in their 2ND AND 3RD trimester. (cART)	IDIs:25 pregnant women	***
9	*Murithi, et al. 2015 [41]	Kenya:	IDIs	HIV + women pregnant receiving care in a PMTCT clinic. (specific short course antiretroviral regimen not mentioned)	IDIs:15 pregnant women	**
10	Elwell, 2016 [43]	Malawi: Urban and rural.	IDIs and FGDs	HIV + pregnant women in a PMTCT programme at four HFs: Community leaders: HCWs. (cART)	IDIs(25 women,19HCWs and 32 community leaders);FGDs(53 women, 32 community leaders)	***
11	*Granato, et al. 2016 [78]	Cote d’ Ivoire: Urban and Rural.	IDIs	HIV positive pregnant women enrolled in PMTCT services attending 30 HFs. (cART)	30 key informant interviews	****
12	Katirayi, et al., 2016 [56]	Malawi: Urban, peri-urban and rural.	IDIs and FGDs	HIV + pregnant and postpartum women enrolled in a PMTCT programme: HCWs. (cART)	39IDIs (19 pregnant women and 20 postpartum women): 16 FGDs (4 pregnant women, 4 HCWs, and 8 postpartum women).	****
13	Kim, et al. 2016 [44]	Malawi: Urban and rural.	IDIs	HIV + pregnant women and postpartum attending ANC at four HF offering ART services. (cART)	65IDIs	****
14	*Napua, et al. 2016 [40]	Mozambique: Urban	IDIs and FGDs	HIV + women pregnant registered in 6 high volume HFs providing PMTCT and ART services: HCWS. (cART)	49 IDIs (8 at 5 sites, and 9 at 1 site): 12 FGDs (ANC patients: 1 FGD per site with 5–10 participants, and with HCWs (1 per site with 6–9 participants).	****
15	O’Gorman, et al. 2010 [55]	Malawi: Rural	IDIs and FGDs	Ante/post-natal women, fathers, grandmothers, TBAs, health workers, community leaders. (specific short course antiretroviral regimen not mentioned)	26 interviews(4 antenatal women, 5 grandmothers, 4HCWs, 5 traditional birth attendants, 3 fathers, 3 church leaders, 2 chiefs): 5 FGDs in total(29 antenatal women in 3 FGDs, 6 postnatal women in 1 FGD, 9 fathers in 1 FGD.	****

*Qualitative aspect of a mixed method study: S/N also indicate study number, the sequential order of articles on this table and on Table 1; differs from the bibliographic reference number: *QAR* Quality assessment rating of studies, *IDI* In-depth interviews, *FGD* Focus group discussion, *Narratives* Narrative structuring, *TBA* Traditional birth attendants, *HCWs* Health care workers, *NC* Antenatal clinic, *HF* Health facility, *PMTCT* Prevention of mother to child transmission of HIV, *ART* Antiretroviral therapy, *cART* Combination antiretroviral therapy(triple drug combination). Quality assessment rating of studies: using the mixed methods appraisal tool an overall quality score of (*) in one of the four methodological assessment area represent 25%; (****) = all four criteria met

### Quality of studies

Tables 3 and 4 show an overview of the quality ratings of studies. When the MMAT tool was applied, 1 of the 11 randomized controlled trials (RCTs) met the 4 criteria with a score of 100%. Among the 31 quantitative non-randomized studies, 18 had a score of 100%. Of the 15 qualitative studies, 9 had a quality rating score of 100%.

### Enablers and barriers of adherence

#### Patient related factors

##### Socio-demographic factors

The most commonly investigated socio-demographic factors included maternal age and education. A number of the quantitative studies (2 RCTs and 11 observational studies) found no association between maternal age and adherence to medication [10, 20–31]. Three observational studies reported a significant association between maternal age and ART adherence [32–34] while a cross sectional study conducted in Nigeria found that pregnant women aged 40 years and older, were less likely to adhere to ART compared with younger women [34]. On the other hand, a cohort study conducted in Malawi demonstrated that women aged 15–29 years were less likely to adhere to ART during pregnancy [32] compared with older women. Overall, the studies showed inconsistent results regarding the effects of maternal age on medication adherence during pregnancy.

Education was the second most investigated socio-demographic factor. Four observational studies reported that a low educational level was significantly associated with maternal non-adherence to medication, in particular, not completing secondary education [29, 35, 36] or primary education [34]. One study also showed that the husbands’ level of education was significantly associated with maternal non-adherence to ART [34]. However, a longitudinal study conducted in Uganda found that mothers with education above secondary level were significantly less likely to accomplish 95% adherence [10]. An inability to read and write in the primary language was also found to be significantly associated with non-adherence in a clustered RCT conducted in Zambia [37]. Studies investigating education showed lack of completion of secondary schooling adversely affected medication adherence. However, pregnant women with high levels of education beyond secondary school also showed low levels of adherence due to their desire to make independent decisions about their medication-taking behavior.

##### Women’s knowledge about human immunodeficiency virus and mother to child transmission of HIV

Two qualitative studies that utilized in-depth interviews and focus groups provided insights about how mothers’ knowledge and beliefs impacted on adherence to ART. A study carried out in South Africa highlighted how some women, because of misinterpretation of information they received, took incorrect dosage of their medication despite counseling being conducted in the local language [38]. In a Ugandan study, participants believed that they had sufficient knowledge about HIV/AIDS and ART to continue or terminate their treatment themselves, without following their prescribers’ intent [39]. In two cross sectional studies conducted in rural and urban settings, one in Nigeria and the other in South Africa, women’s knowledge about MTCT was significantly associated with maternal adherence to ART medications [27, 31]. Being well informed about HIV and prevention of MTCT among women appeared to encourage adherence to ART during pregnancy, although misinterpretation of health professionals’ counseling led to inappropriate decisions made by pregnant women about their medication-taking activities.

##### Psychological factors

Qualitative research provided useful insights on the impact of the emotional and psychological state of pregnant women following a diagnosis of HIV. In-depth interviews and focus groups conducted in Mozambique showed that pregnant women were not emotionally or socially ready to continue lifelong treatment due to high levels of HIV related stigma in their communities [40]. Similarly, in-depth interviews and focus groups conducted in Kenya revealed that the shock experienced from being diagnosed as HIV positive remained a barrier to pregnant women’s ability to initiate and adhere to ART [30, 41]. Refusal by pregnant women to accept their HIV sero-status was also identified as an obstacle to ART adherence, because of fear that family members and friends may isolate or stigmatize them [42]. However, interviews conducted in Cote d’ Ivoire, in western Africa, revealed that the motivation to protect their unborn baby was more pronounced in mothers who had previously lost young children to HIV. They were determined to prevent losing another child to the disease by seeking to maintain good adherence to ART during pregnancy [42]. Likewise, interviews conducted in Kenya showed that HIV positive women who longed for motherhood, viewed taking and adhering to ART during pregnancy as a way of bearing HIV negative children [41]. For some women in Malawi, the desire to prevent HIV from infecting their unborn children, to maintain their health, and to be able to work, motivated them to adhere to ART [43]. In essence, the shock of receiving an HIV positive result during pregnancy had the potential to unsettle the emotional state of women, thereby interfering with adherence negatively. Nevertheless, the desire to ensure that the unborn child is protected from HIV infection, was an important motivator for women to adhere to ART during pregnancy, especially for those who had already lost a child to the disease.

##### Women’s belief system

Women’s belief systems, as demonstrated by the value placed on religion and their role in taking on family responsibilities, also influenced medication-taking behavior. Interrupted personal routines associated with been away from home, attending church, a funeral or a party, were reasons given for missed doses of ART in a study conducted in South Africa [38]. Religious belief was reported as a barrier to adherence in a qualitative study [44], whereas a case-control study showed no association between religious affiliation and adherence in HIV positive pregnant women [29]. A qualitative study conducted in Kenya provided insight on how the cultural environment comprising women’s customs, beliefs and behaviors, influenced the use of traditional medicine together with ART [45]. A sub-analysis of a RCT and an observational study in Zambia and Congo, Brazzaville showed that use of traditional medicine was significantly associated with non-adherence to ART during pregnancy [46, 47]. Traditional medicines were sometimes used as a substitute for ART as women sought to find what they perceived to be more effective, or safe alternatives. Religious activities appeared to have a mixed influence on medication adherence.

#### Patient condition related factors

##### Women’s obstetric and intrapartum history

Two cross sectional studies showed that multi-parity (4th baby and above) was associated with non-adherence in HIV positive pregnant women [34, 36] although this association was not demonstrated in other similar studies [21, 24, 25, 30, 31]. One possible reason could be related to studies being conducted in different countries. In two studies that were conducted in Nigeria, the research was undertaken in two different regions of Nigeria, which had distinct socio-cultural outlooks.

A qualitative study using in-depth interviews demonstrated that women who had previous experience with prevention of MTCT were better prepared and more compliant with taking ART in their current pregnancy [41]. However, observational studies conducted in South Africa and Nigeria did not show an association of previous maternal exposure to prevention of MTCT and adherence to ART [31, 35].

Diagnosis of HIV either immediately before, or during, pregnancy was found to be a predictor of non-adherence to ART. A cohort study in Zambia showed that women newly diagnosed with HIV during pregnancy were more likely to be non-adherent than those with a known HIV status before pregnancy [22]. An association with a positive HIV diagnosis during pregnancy with non-adherence to ART was also found in a cross sectional study conducted in Nigeria [34]. In Burkina Faso, a significant association of knowledge of HIV status immediately before pregnancy and adherence to ART was found in a cohort study [33]. Consequently, the knowledge of a positive HIV diagnosis immediately before and during pregnancy was a major predictor of non-adherence in women taking ART. On the other hand, previous experience with prevention of MTCT was shown to improve adherence among women during their current pregnancy.

##### Disease progression

Opportunistic infections occur from progressive damage to the immune system. Up to that point, there is an asymptomatic phase of the disease where the person may not experience any symptoms. During this asymptomatic phase, women live in denial and may not perceive the need to commence or continue ART. In a study involving in-depth interviews, Malawian HIV positive pregnant women were reluctant to initiate ART because they were not experiencing symptoms related to the disease [44].

Two observational studies conducted in Ethiopia and Malawi found the WHO clinical staging of HIV in women was associated with adherence to ART [32, 48]. The WHO clinical stage 1 signifies women are asymptomatic, or have persistent generalized lymphadenopathy, while those in the clinical stage 4 signifies severe signs and symptoms of the disease. In Malawi, women in the clinical state 3 were less likely to adhere to treatment than those in WHO clinical stage 1 [32]. Similarly, Ethiopian women in the clinical stage 1 or 2 were also found to be more likely to be adherent than women in the clinical stage 3 or 4 [48]. Evidence suggests that non-adherence in advanced HIV disease may be as a result of increased opportunistic infections and HIV complications requiring many medicines to treat these conditions [49].The immunological status (CD4+ cell count) of women was also linked with adherence to ART in pregnancy [48]. This is because as the CD4+ cell count increased, adherence to ART improved. It appears as though there are mixed results in terms the influence of disease progression on medication adherence.

#### Therapy related factors

##### ART side effects and efficacy of therapy

Two qualitative studies based on in-depth interviews reported the influence of ART side effects on adherence during pregnancy. The first study, conducted in Cote d’Ivoire, highlighted that pregnant women complained of side effects, such as tiredness and weakness, but these effects did not prevent them from continuing with their medication [42]. Side effects were also a reported barrier to ART adherence in the second study, which was carried out in Malawi. Almost all women experienced side effects such as dizziness, hallucinations, nightmares, nausea and vomiting. Results from this study further revealed that women reported worsening of ART side effects in the absence of food. Some stopped taking their ART occasionally due to their inability to consume food from a lack of availability. The authors posited that women had poor adherence during times of food scarcity and when side effects were too severe to manage [44]. A cross analytical study conducted in the Democratic Republic of Congo found no association between ART side effects and adherence [46].

Two qualitative studies, using in-depth interviews, reported on perceived effectiveness of ART among women during pregnancy, thus facilitating adherence to medication [41, 42]. Previous successful experiences of mothers taking ART that resulted in giving birth to healthy children, served as a source of confidence, which facilitated adherence to medication. Side effects of ART, coupled with food shortage, appeared to be major obstacles to adherence during pregnancy. The perceived efficacy of ART also facilitated adherence to ART among the women.

#### Social and economic related factors

##### Financial difficulty, income generating activities and occupation

Four qualitative studies, utilizing in-depth interviews and focus groups, showed that limited finances, specifically relating to the cost for transportation to the health care clinics and hospitals, was a barrier to adherence. Financial constraint emerged as the greatest hindrance to taking ART in a RCT conducted in Uganda [39]. In that study, the high cost of transportation from the pregnant women’s home to the clinic was the most prevalent barrier to enrolling in the preventative MTCT Plus Program and adherence to ART [39]. Other qualitative studies [28, 30, 44] conducted in Swaziland, Kenya and Malawi also reported transportation barriers due to lack of finances. However, cross sectional and longitudinal studies, carried out in Democratic Republic of Congo and Uganda, found no association between lack of finances for transportation to the clinic and medication adherence [10, 46]. Financial constraint to buying food was also a major barrier to adherence in many women, as revealed in three qualitative studies [38, 39, 44]. As a result of financial limitations, women were unable to purchase sufficient food to ensure they took ART with food as they were concerned about taking ART on an empty stomach.

Several observational studies and one RCT reported no association between income generating activities, or people’s occupation on adherence to ART during pregnancy [10, 21, 26, 30, 31, 35, 36]. However, one cross sectional study conducted in Nigeria reported an association of occupation and non-adherence. The study reported that women who were artisans, or full-time housewives, were more likely to be non-adherent compared with women working in a trade or the public service. Women who were students were adherent to their ART [34]. The poor financial state of women proved to be a major obstacle to ART adherence in terms of consuming enough food to take their medication at the required time, and in terms of inhibiting transportation to scheduled ART refill appointments.

##### Women empowerment

The issue of women empowerment was examined during in-depth interviews carried out in Uganda, where women reported economic dependence on their husbands. These women were unable to independently determine when to buy food or related household items that could assist with medication adherence [39]. It was posited that a lack of female economic empowerment was a limiting factor over treatment-seeking behavior and ability to initiate and adhere to ART [39]. A cohort study undertaken in South Africa demonstrated that not receiving financial support from a partner, or a husband, was significantly associated with incomplete adherence in women receiving ART during pregnancy [35].

##### Domestic and partner violence

In an in-depth interview study conducted in South Africa, domestic violence, which included both actual physical abuse or threatening behavior, was mentioned by many women as interfering with adherence to ART [38]. Intimate partner violence was significantly associated with non-adherence to antiretroviral drugs during pregnancy, especially sexual violence, as reported in a cross-sectional study undertaken in Zambia [21].

##### Male partner involvement in antenatal clinic attendance

Male involvement was associated with maternal nevirapine and ART adherence in cross sectional studies conducted in Ethiopia and South Africa [48, 50]. By contrast, lack of male involvement was not associated with ART adherence in a longitudinal study conducted in Uganda [10]. One qualitative study, using in-depth interviews, reported lack of male involvement as part of the overall challenges facing the delivery of a comprehensive MTCT service in Swaziland [28].

##### Disclosure of HIV infection status

Qualitative studies highlighted the extreme difficulties that most pregnant women faced regarding disclosure of HIV status to partners, family and friends. An in-depth interview study in Kenya described HIV disclosure to spouses to be a dreadful experience for women because of negative consequences on the women’s marriage, such as violence and accusations of infidelity, as well on their health and well-being, including taking ART [41]. One study, using focus groups and interviews in Uganda, reported that non-disclosure was a common barrier to enrolling in an MTCT program and continuing ART because of fear of blame, domestic violence, divorce, abandonment and loss of economic support that might follow. The study also highlighted the fear of HIV positive pregnant women in exposing their status to their partners by suggesting the use of safe sex practices [39]. Some women faced outright obstructive behaviors from their spouse, such as throwing away their pills after HIV disclosure, as revealed during in-depth interviews in a Malawian study [44]. A similar study in South Africa reported women being afraid of disclosing their HIV status to family members because of fear of discrimination [38]. In studies conducted in Malawi and Mozambique, the fear of disclosure to partners and spouses was highlighted as a barrier [40, 43].

In qualitative studies undertaken in South Africa and Kenya, as a consequence of fear of disclosure, many women resorted to hiding their ART in their homes, away from partners and family [38, 45]. Fear of disclosure also presented a problem when hidden medications needed to be brought to the health facility for pill counts [38]. However, there are studies describing the supportive roles of partners and family members with respect to adherence of medication when women disclosed their HIV status [45, 51]. Quantitative research conducted across several of the sub-Saharan African countries, including Nigeria, Rwanda, Ethiopia, Kenya, Tanzania, Zambia, and South Africa demonstrated an association between disclosure to partners, family and friends and maternal adherence to ART. Seven observational studies and a RCT showed that disclosure was associated with good maternal adherence [21, 25, 27, 30, 48, 52–54] while non-disclosure was associated with non-adherence in two observational studies [29, 34]. A cross sectional study in Nigeria showed an association between disclosure of a partner’s HIV status and women’s adherence to medication, particularly if the partner was also HIV positive [34]. Fear of disclosure of HIV infection status and the negative consequences that came with such disclosure was a major barrier to ART adherence during pregnancy. However, male partners who were HIV positive and those who accepted the HIV status of their female partners tend to be supportive of their spouse’s adherence to ART.

##### HIV related stigma and discrimination

The initiation, adherence and retention of ART were largely influenced by stigma and fear of discrimination. Qualitative studies showed the complexities around the issue of stigma and women’s adherence to ART. In a study in Malawi where focus groups and in-depth interviews were conducted, women reported shame as the reason why they would not return to the MTCT clinic after testing HIV positive. For those who continued treatment, they devised means of concealing their HIV status in order to avoid stigma, such as travelling to distant health care facilities to ensure anonymity and hiding their ART [43]. Research conducted in Malawi, showed the fear of stigma hindered women from taking their nevirapine at home as prescribed [55]. However, in a recent Malawi study involving in-depth interviews, while stigma was acknowledged by HIV positive pregnant women, there were few reports of overt discrimination and only one patient stopped ART because of stigma [44]. Similarly, women interviewed in Kenya expressed fears of being rejected by their partners and causing shame to their loved ones. They therefore travelled long distances to attend MTCT clinics, located outside of their local communities [41]. Likewise, in another Kenya study, women discussed fear of stigma if their HIV infection status was disclosed. The women recounted that they were able to attend their clinical appointments and adhered to taking their medicines because the community was unaware of their HIV status [45]. A qualitative study also reported stigma as a major challenge to delivering comprehensive MTCT services in Swaziland [28]. Nevertheless, most pregnant women recognized that HIV/AIDS stigma was prevalent within their communities, but the women claimed that the community view of HIV infected persons had no bearing on their decision to commence or continue ART, as shown in an interview study in Uganda [39]. In essence, evidence suggests that stigma and discrimination are strong barriers to ART adherence during pregnancy.

##### Sharing or stealing medications by partners, friends and relatives

In interviews conducted in South Africa, women reported that HIV-infected relatives stole their ART from their home. They attributed the discrepancies in their ART-taking behavior to theft of the medications, hence limiting their adherence [38]. Focus group research in Kenya also highlighted how women shared their medications with partners, which led to poor adherence to ART [30].

##### Positive outlook of known patients living with HIV in the community, place of residence and social support systems

An important facilitator identified during focus groups and interviews, was women observing positive outcomes in the health and wellbeing of other women taking ART within communities in Kenya and Malawi [41, 43, 56]. In a cohort study conducted in Uganda, the place of residence comprising a village environment where individuals were exposed to large amounts of information about prevention of MTCT, was associated with good maternal adherence to nevirapine. In this study, women were exposed to information about prevention of MTCT [57]. Conversely, in a cross sectional study conducted in Ethiopia, living in a rural or urban setting was not associated with adherence to ART. In this study, women were not exposed to extensive information about prevention of MTCT [48]. Attending a support group, and having partner support were found to be positively associated with maternal adherence to medication in observational studies carried out in Ethiopia [48], South Africa [50, 52] and Nigeria [31].

#### Health care team and health system related factors

##### Number of antenatal care visits and clinic attendance

Quantitative research conducted in Zambia demonstrated that the odds of ingesting a single dose of nevirapine was greater among pregnant women who attended three or more antenatal care visits compared with women who attended two or fewer visits [58]. A significant association was found between more than two antenatal care visits and adherence to a single dose of nevirapine in a study completed in South Africa [27]. A similar study involving attendance by pregnant women of at least one antenatal care clinic was also found to be significantly associated with adherence to ART in Ethiopia [48]. Health facility delivery and term delivery was also linked with maternal adherence to a single dose of nevirapine in a cross sectional study conducted in South Africa [27].

##### Staff related factors

A subgroup analysis of one RCT demonstrated that structured counseling by health care workers was a facilitating factor for pregnant women to adhere to their medication. A cross sectional study in Ethiopia documented that mothers who were given accurate counseling on the appropriate intake of ART had 4.7 times higher odds of adhering to option B+ care and support than women who were not correctly counseled [48]. A similar study conducted in South Africa showed evidence of association between adherence to single nevirapine dose and the quality of HIV counseling [27]. Qualitative studies utilizing focus groups and interviews indicated the benefits of health care providers’ counseling to help pregnant women optimize the benefits of ART. A study in Tanzania showed nurses provided high quality psycho-social support to women that resulted in good adherence to antiretroviral (ARV) drugs [59]. Pregnant women in studies undertaken in Uganda and Cote d’Ivoire also reported positive interactions with antenatal care staff that facilitated adherence to ART [42, 51].

Nevertheless, qualitative research has also described the negative impact of health care workers’ attitude and behavior on the ability of women to initiate, adhere and continue ART. Focus groups conducted in Kenya revealed that mistreatment by midwives was one of the reasons women reported difficulties in the use of zidovudine [30]. Women feared that midwives would not provide the required assistance during delivery if they knew their HIV infection status. During focus groups and interviews conducted in Malawi and Uganda, poor interaction with health workers was identified as a major barrier to ART adherence [39, 43]. Poor interactions included disrespectful behaviors such as shouting and making rude comments. Well-structured and accurate counseling on the benefits of ART, good rapport and provision of effective psycho-social support by health professionals were major enablers of ART adherence during pregnancy. Conversely, negative attitudes of health professionals towards women were barriers to ART adherence.

##### Resource, infrastructure, service related factors and the supply chain management system

Qualitative studies using focus groups and interviews showed that prolonged counseling to initiate ART treatment, or prevention of MTCT prophylaxis, was perceived by health care providers as being counterproductive because it increased the nonattendance rate and disease progression among pregnant women [28]. In addition, long waiting times, poor counseling due to short contact time between health care providers and women all impacted negatively on the ability of mothers to continue and adhere to ART in Swaziland, Uganda and Tanzania [39, 59]. Lack of privacy and not trusting health workers to keep women’s HIV status confidential were cited by many women as reasons for not initiating or continuing ART [28, 59]. Unpredictable shortages or delayed re-supply of ART were found to reduce patient adherence in Swaziland, Cote d’Ivoire and Tanzania [28, 42, 59].

## Discussion

### Key findings and programmatic implications

The review identified barriers and enablers linked to patient related factors, condition related factors, therapy related factors, social and economic related factors, and health care team and health system related factors. At the patient level, psychological factors such as shock and denial following HIV test results, were prominent barriers to ART adherence. Patient socio-demographic factors such as mothers’ age and maternal education were also frequently reported barriers of adherence during pregnancy. Social and economic factors also affected medication adherence behavior of most women during pregnancy. Stigma about the HIV condition, cost of transportation, nutritional deprivation and a woman’s disclosure or non-disclosure of her HIV status were barriers to pregnant women’s adherence to prescribed ART. Health care related factors, such as negative attitudes of health workers, unpredictable shortages or delayed re-supply of medications, long waiting times and confidentiality issues at health facility were also major barriers. Enablers included well-structured and accurate counseling on the benefits of ART, good rapport and provision of effective psycho-social support by health professionals during pregnancy.

This review uncovered the patients’ condition related factors as important reasons affecting ART adherence in pregnancy [15, 60–62]. Furthermore, the review has shown that knowledge of HIV status, either before or during pregnancy, was significantly associated with medication adherence. Women who knew their HIV status before pregnancy demonstrated good adherence while women who found out their HIV infection status during pregnancy were linked with non-adherence to ART. Thus, the knowledge of HIV status before pregnancy is crucial for disease acceptance and management [33]. Disclosure of HIV status was closely tied to women’s fear and stigma of HIV, which requires the need for innovative approaches that engage individuals and communities to create an enabling environment for prevention of MTCT. Increased and tailored counseling for women, partners and family members is needed to reduce stigma and improve disclosure. Furthermore, greater understanding is needed about the historical circumstances and social family situations comprising the family unit affected by HIV. There is also the need to intensify early diagnosis of HIV in women and men within communities based on the 90–90-90 targets. Diagnosis of HIV in women before pregnancy will help to minimize the emotional burden of being diagnosed HIV positive during antenatal visits.

Intimate partner violence of a sexual, emotional and physical nature was strongly linked with non-adherence to ART during pregnancy. There is a need for urgent intervention to address the risk factors associated with intimate partner violence and domestic violence at the individual and relationship level. Issues affecting intimate partner violence, such as low levels of education and male dominance that fuels the norm of gender inequity, should be addressed [63]. Community awareness is required to facilitate greater understanding about intimate partner violence. Promoting an enabling environment is needed within antenatal settings to facilitate male engagement about enhancing positive relationship building. To encourage male partner attendance, this can be achieved by the introduction of flexible times for antenatal clinic attendance to accommodate the work commitments of male partners. Male partner attendance in antenatal clinics will help to promote an ethic of responsibility among men for the health and well-being of their pregnant partner.

Assessments of methodological quality reveals strengths and weaknesses of the studies included in the review. There was possible bias relating to allocation concealment in many of the RCTs [20, 37, 38, 40, 47, 58, 64–67]. In these studies, the method used to conceal the allocation sequence was not described or described in insufficient detail to determine whether intervention allocations could have been foreseen in advance of, or during, enrolment. Of the eleven included RCTs, it was only in one study where investigators enrolling participants could not foresee assignment of women to a particular treatment arm [30]. Missing outcome data due to attrition from drop outs and loss of data were not accounted for in some of the studies during analysis [20, 38, 47, 58, 65]. Missing outcome data due to participant attrition raises the possibility that the observed effect estimate is biased [68]. In addition, many of the quantitative outcome measurements such as medication adherence [22, 27, 29, 31, 34, 36, 46, 48, 52–54, 57, 66, 69, 70] were based on women’s and health workers self-reports, which could have led to recall bias. There were some studies that reported medication adherence measurements based on objective methods, such as pill counts and pharmacy refill records [10, 20, 25, 32, 35, 38, 40, 64, 67, 71]. In a few qualitative studies, appropriate considerations may not have been given to how findings related to the researchers’ influence, such as through their interactions with participants [42, 43]. In situations where investigators who were not native to sub-Saharan Africa were involved in conducting interviews with local individuals, it is possible that these investigators lacked understanding and appreciation of the social structure of the local context. The historical customs and culture of individuals could affect communication processes, and greater clarity and reflections about these aspects would have enhanced understanding about the investigators’ perspectives, and the ways in which these perspectives affected study results.

The findings from this review allude to two major areas where urgent attention is needed to improve adherence to ART during pregnancy. First, the challenges of fear of disclosure of HIV status by women should be addressed. As demonstrated in this review, fear of disclosure to partners, family and the community was a major barrier to adherence during pregnancy and uptake of MTCT services [15, 60, 61]. According to the WHO, pregnant women in sub-Saharan Africa have the lowest rate of HIV status disclosure to partner in the world [72]. Second, HIV stigma and discrimination remain potent barriers to ART adherence for most women at family and community levels. Thus, the findings in this review has far reaching implications for the UNAIDS goal of achieving an AIDS free generation and ending the HIV epidemic by 2030 [73, 74], since the ability to accomplish virtual elimination of MTCT depends not only on the availability and accessibility to ART, but also on the ability of women to take their medications as prescribed.

### Limitations of the review

The review has some limitations as it did not explore the entire spectrum of prevention of MTCT adherence to ART. This review was limited to adherence to ART during the antepartum and intrapartum period. The postpartum period was not included, however, many of the enablers and barriers to ART adherence identified in this review may have implications for the postpartum period. The reasons for potential heterogeneity in the quantitative studies may be result of the different definitions and threshold of adherence, different measurements of adherence, especially self-reporting adherence measures that may be subject to recall bias.

## Conclusions

This review revealed several barriers and enablers of adherence among pregnant women taking ART in sub-Saharan Africa. Evidence suggests that stigma and discrimination at community levels are strong barriers to ART adherence during pregnancy. In addition, the fear of disclosure of HIV infection status and the negative consequences that came with such disclosure emerged consistently across a range of settings in sub-Saharan Africa as a major barrier to ART adherence. One major enabler of adherence in women taking ART is the knowledge of HIV status prior to being pregnant. Thus, the knowledge of HIV status before pregnancy is crucial for disease acceptance and management.

## Abbreviations

ANC: Antenatal Clinic
ART: Antiretroviral Therapy
DRC: Democratic Republic of Congo
HIV: Human Immunodeficiency Syndrome
MMAT: Mixed Methods Appraisal Tool
MTCT: Mother to child transmission of HIV
RCT: Randomized Controlled Trails
UNAIDS: United Nations Joint Programme on HIV/AIDS
WHO: World Health Organization
